# Phytochemical Characterization Utilizing HS-SPME/GC-MS: Exploration of the Antioxidant and Enzyme Inhibition Properties of Essential Oil from Saudi *Artemisia absinthium* L.

**DOI:** 10.3390/ph17111460

**Published:** 2024-10-31

**Authors:** Hanan Y. Aati, Hala A. Attia, Arwa S. Alanazi, Luluh K. AL tamran, Juergen K. Wanner

**Affiliations:** 1 Department of Pharmacognosy, College of Pharmacy, King Saud University, P.O. Box 2457, Riyadh 11451, Saudi Arabia; 2 Department of Pharmacology and Toxicology, College of Pharmacy, King Saud University, P.O. Box 2457, Riyadh 11451, Saudi Arabia; hsalem@ksu.edu.sa; 3 College of Pharmacy, King Saud University, P.O. Box 2457, Riyadh 11451, Saudi Arabia; 441200341@student.ksu.edu.sa (A.S.A.); 441200557@student.ksu.edu.sa (L.K.A.t.); 4 Kurt Kitzing Co., Hinterm Alten Schloss 21, D-86757 Wallerstein, Germany; juergen.wanner@kurtkitzing.de

**Keywords:** essential oil, HS-SPME, HD, enzyme inhibitions, NADPH oxidase, GC-MS, antioxidants, in silico molecular docking, BOILED-Egg model

## Abstract

**Background/Objectives:** This study aimed to analyze the chemical composition and biological activities of *Artemisia absinthium* L. essential oil, focusing on its antioxidant and enzyme inhibition (α-amylase and urease) properties. Additionally, in vitro pharmacokinetic and pharmacodynamic evaluations were conducted through in silico molecular docking and BOILED-Egg models to assess its therapeutic potential and its potency in treating oxidative-stress-related diseases. **Methods:** The essential oil was isolated by the hydrodistillation (HD) of fresh plant material, and volatiles released from dried plant material were sampled via headspace solid-phase microextraction (HS-SPME), followed by a phytochemical profiling analysis through the GC-MS tool. Antioxidant capacity was assessed using DPPH, ABTS, FRAP, and nitric oxide scavenging assays, while enzyme inhibition activities were tested against α-amylase and urease. Molecular docking and BOILED-Egg models were used to evaluate compound interactions with NADPH oxidase and predict pharmacokinetic behavior, respectively. **Results:** HS-SPME and HD yielded 46 and 25 compounds, respectively, primarily terpenoids represented by camphor (26.4%) and cis-davanone (18.0%) in HS-SPME, while in the HD essential oil, cis-davanone (60.2%) and chamazulene (10.8%) were most prevalent. The antioxidant assays showed a strong activity, with a total antioxidant capacity of 821.8 mg ascorbic acid Eq/gm. The essential oil inhibited urease by 86.7% and α-amylase by 81.8%. Molecular docking showed strong binding affinities with NADPH oxidase, supporting the antioxidant results. **Conclusions:** *A. absinthium* EO demonstrated potent antioxidant and enzyme inhibitory activities, suggesting its therapeutic potential for treating enzyme-related disorders like diabetes mellitus and its possible use as a cure for many oxidative-stress-related diseases, thus validating the folkloric use of this plant.

## 1. Introduction

The prevalence of diseases, particularly those linked to oxidative stress and enzyme dysfunction, is a significant global health concern. Contemporary illnesses such as diabetes, cardiovascular disease, cancer, and neurodegenerative disorders have been strongly associated with free radicals and enzyme imbalances, such as α-amylase and urease [1,2]. These free radicals lead to oxidative stress, which damages cells, proteins, and DNA, contributing to the progression of many degenerative diseases [3]. Enzyme-related conditions, particularly those involving α-amylase and urease, further exacerbate the pathology of diseases like diabetes, obesity, and ulcers [4].

Natural remedies have been extensively investigated for their curing potential in combating such unwanted diseases [5]. Unlike synthetic drugs, which can have adverse effects, natural compounds offer a safer alternative due to their reduced toxicity and improved biocompatibility [6]. Essential oils (EOs) are among the most significant secondary metabolites of plants, known for their remarkable range of biological activities, possessing antimicrobial, antioxidant, and anti-inflammatory properties [7]. These essential oils, rich in bioactive compounds, exhibit synergistic effects that enhance their therapeutic efficacy [8]. When used in combination, the various compounds in essential oils can work together to improve their biological activity compared to a single isolated component [9].

Extracting essential oils from natural sources is crucial for maintaining the integrity of their bioactive compounds [10]. Traditional methods, such as steam or hydrodistillation, can sometimes degrade volatile components [11]. In contrast, techniques like headspace solid-phase microextraction (HS-SPME) offer distinct advantages in EO sample preparation [12]. It is a solvent-free, rapid, and efficient technique that provides samples from dried plant material without altering their chemical structure [13].

The Asteraceae family includes a wide variety of plants known for their therapeutic effects, especially in treating inflammation, infections, and chronic diseases [14]. Belonging to the family Asteraceae, Artemisia species are renowned for their medicinal properties [15,16]. For centuries, various Artemisia species have been employed in traditional medicine to cure a wide range of health problems, including digestive disorders and skin conditions [17]. *Artemisia absinthium*, commonly known as wormwood, has a rich history in folkloric medicine, particularly in regions like Saudi Arabia, where it has been utilized for its analgesic and antidigestive properties, as well for its anti-inflammatory potency [18]. Locally, it is known as ‘Boaitheran; Shih’.

Several volatile compounds have been identified as key constituents in *A. absinthium*, with varying chemotypes reported depending on the region and growing conditions. In Tunisia and Algeria, camphor, β-selinene, β-thujone, trans-sabinene hydrate, and chamazulene were found to be the major constituents, while chamazulene, camphor, davanone, and ethyl (E)-cinnamate were representative of the Ethiopian species. In the Indian and Iranian species, borneol, methyl hinokiate, and isobornyl acetate as well as, chamazulene, β-pinene, α-pinene, sabinene, α-phellandrene, and p-cymene were the major constituents in the essential oils, respectively [18]. Besides its volatile components, the plant also contains phenolic acids, isoflavonoids, tannins, and flavonoids. Previous pharmacological reports have confirmed that this plant exhibits antimalarial, antimicrobial, anticancer, and hepatoprotective properties [18,19,20].

Preceding studies on *A. absinthium* EO have primarily focused on composition analyses and biological activities, revealing its potential for antioxidant, antimicrobial, anti-inflammatory, antidepressant, antiulcer, and cytotoxic activities [19] due to its active constituents, such as thujone, myrcene, camphor, and linalool. These compounds are notable for their antimicrobial, antifungal, and antioxidant effects. For instance, thujone, a prominent monoterpene in the EO, has demonstrated strong antimicrobial properties, which are valuable in combating pathogens resistant to conventional antibiotics [17]. Additionally, camphor and myrcene contribute to the anti-inflammatory effects, making the essential oil useful in reducing inflammation and managing pain [17,19]. Studies also indicate that *A. absinthium* EO possesses anticancer potential, with compounds like camphor and linalool showing cytotoxicity against various cancer cell lines, including liver and breast cancer cells, by promoting apoptosis [19].

Apart from these, the antioxidant properties are significant, particularly due to the presence of phenolic compounds and flavonoids in *A. absinthium* extracts, which help in neutralizing free radicals. This activity is further supported by constituents like camphene and cadinene, which enhance cellular protection mechanisms against oxidative stress, potentially reducing the risk of chronic diseases [18,19]. Thus, the essential oil of *A. absinthium*, owing to its bioactive compounds, shows a diverse therapeutic potential that is considered more potent and specific in its activity.

However, research on essential oil prepared from Saudi-origin *A. absinthium* using techniques like HS-SPME has been limited [20]. The current work is the first of its kind to isolate essential oil from *A. absinthium* collected from the lands of the Kingdom of Saudi Arabia using HS-SPME. Since the Kingdom of Saudi Arabia is characterized by the diversity of its climate and geography, which is reflected in the variety of its flora and consequently the diversity of their compounds, we conducted this study to compare our findings on EO composition with previous reports, aiming to highlight the significance of factors such as temperature, soil composition, and environmental conditions in influencing variations in EO yields and physical properties. These differences were observed not only between the same species growing in different parts of the world but also within the same species growing in various regions or provinces within the same country. This comparison points to an important insight regarding EO composition, which could serve as a chemotaxonomic tool. For that, in this study, we isolated EO from the fresh plant material of the titled species using HD, while volatiles released from dried plant material were sampled via HS-SPME. We then compared the compositions via GC-MS analysis. This comparison aimed to demonstrate the influence of compositional variations in the field of chemotaxonomy. Additionally, the current work evaluates the antioxidant and enzyme inhibition activities, employing in silico molecular docking to pinpoint the exact constituents responsible for the observed antioxidant and enzyme inhibition effects, providing a deeper understanding of the essential oil’s therapeutic potential. Nicotinamide adenine dinucleotide phosphate (NADPH) oxidase, a key source of intracellular reactive oxygen species (ROS), has gained significant attention over the years. This enzyme has been linked to various diseases, including neurodegenerative disorders and cancer. Therefore, we selected it for our in silico molecular docking study [21]. Furthermore, the pharmacokinetics (PK) of the EO was assessed by using one in vitro model known as BOILED-Egg to predict the PK behavior for the various EO constituents. Consequently, the study of the EO from a pharmacokinetics (PK) and pharmacodynamics (PD) view will help to optimize the bioavailability and therapeutic index and guide structural modifications while reducing the reliance on animal testing. These types of assays provide early insights into drug interactions, toxicity, and mechanisms of action for effective drug development.

Finally, the findings from this study are significant on a global scale as they pave the way for the development of pharmaceutical compounds derived from natural sources, which offer greater benefits and fewer side effects compared to synthetic drugs. By identifying the bioactive compounds from *A. absinthium* isolated EO and understanding their constituents’ mechanisms of action (PD) and PK behaviors, this research contributes to the growing field of natural product drug discovery and highlights the potential of essential oils in treating enzyme- and oxidative-stress-related disorders [22].

## 2. Results

### 2.1. GC-MS Composition Analysis for Essential Oils Isolated via HD Using Fresh Plant Material and Volatiles Released from Dried Plant Material Using HS-SPME

The GC-MS analysis was used to recognize the phytocompounds available in the EOs. The results showed 46 and 25 peaks of compounds for HS-SPME and HD, respectively, that belong to different chemical classes, i.e., terpenoids, aldehydes, ketones, alcohols, esters, and phenol derivatives, representing 95.6% and 98.5%, respectively, of the total isolated dark blue EO content. The blue color of EO isolated by HD was due to it containing a higher percentage of chamazulene (a bicyclic sesquiterpene derivative), which constituted about 10.8%, while the EO prepared by HS-SPME was 2.8%. Oxygenated monoterpenes were shown to be the major class of constituents in EO prepared by HS-SPME (42.0%), mainly represented by camphor (26.4%), while in HD EO, they represented 13.8%, with camphor constituting 6.3% of this percentage. On the other hand, oxygenated sesquiterpenes represented significantly more than 50% of the content in EO extracted by HD (64.3%), with cis-davanone constituting 60.2%, while in HS-SPME, it was 18.0%.

Monoterpene hydrocarbons comprised 16.0% of the total identified constituents of EO prepared using HS-SPME, represented by limonene (3.3%), camphene (3.0%), and α-pinene (2.3%), while the same class of isolated components from the HD method only comprised 3.9%. Some compounds identified in the EO prepared by HS-SPME were not identified in the HD or only present in minute quantities less than 0.05%, such as (E)-ethyl cinnamate (5.4%), (E)-nerolidol (4.6%), bornyl acetate (4.2%), linalool (3.7%), camphene (3.0%), 4-thujanol (2.3%), and carvone (2.0%). This indicates that a greater number of compounds, belonging to various chemical groups, were identified in the EO prepared using the HS-SPME method.

All the identified compounds are described in Table 1, and the peaks of the identified compounds are presented in Figures S1 and S2; the constituents are listed in order of their elution from the column and retention indices (RIs). The identification of these compounds was achieved by comparing their peak retention indices and mass spectral fragmentation patterns with known compounds in the National Institute of Standards and Technology (NIST) library.

### 2.2. Biological Activity

#### 2.2.1. Antioxidant Potencies of *A. absinthium* EO

2,2-diphenyl-1-picrylhydrazyl (DPPH), 2,20 -azino-bis (3-ethylbenzothiazoline-6-sulfonic acid (ABTS+), ferric reducing antioxidant power (FRAP), nitrite oxide scavenging (NO), and total antioxidant capacity (phosphomolybdenum) (TAC) assays were used to analyze the antioxidant potential of EO. The maximum antioxidant potency was shown by TAC, followed by ABTS, DPPH, FRAP, and NO assays, respectively. The significant finding of the free radical scavenging potency of EO referred to the synergistic effect of bioactive constituents belonging to various chemical classes, especially oxygenated terpenoids. The results of the antioxidant assays are presented in Table 2, and all the results are calculated as mg·Eq·gm^−1^ EO.

#### 2.2.2. Enzyme Inhibition Activities

The enzyme inhibition potential of EO was assessed for its potential application in pharmaceuticals, specifically for its antidiabetic (α-amylase inhibition) and antiulcer (urease inhibition) properties. Acarbose and thiourea were used as positive controls to evaluate enzyme inhibition. The results indicated that EO demonstrated α-amylase inhibition with an efficacy of 81.8%, compared to acarbose’s 90.8%. Similarly, EO showed a urease inhibition effect of 86.7%, close to thiourea’s 91.7%. The enzyme inhibition results for EO are summarized in Table 3.

### 2.3. In Silico Molecular Docking Study (NADPH oxidase Enzyme Inhibition), (In Vitro Pharmacodynamics Study)

**Figure 1:**
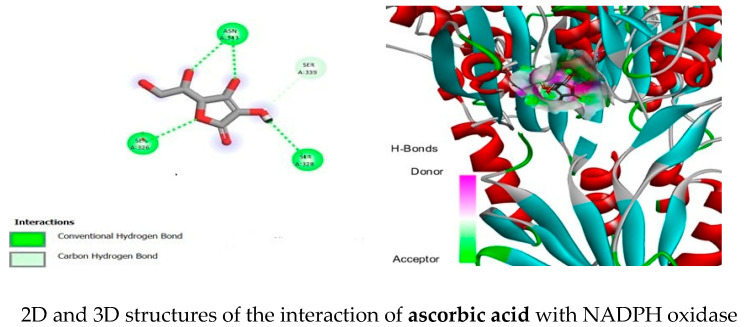

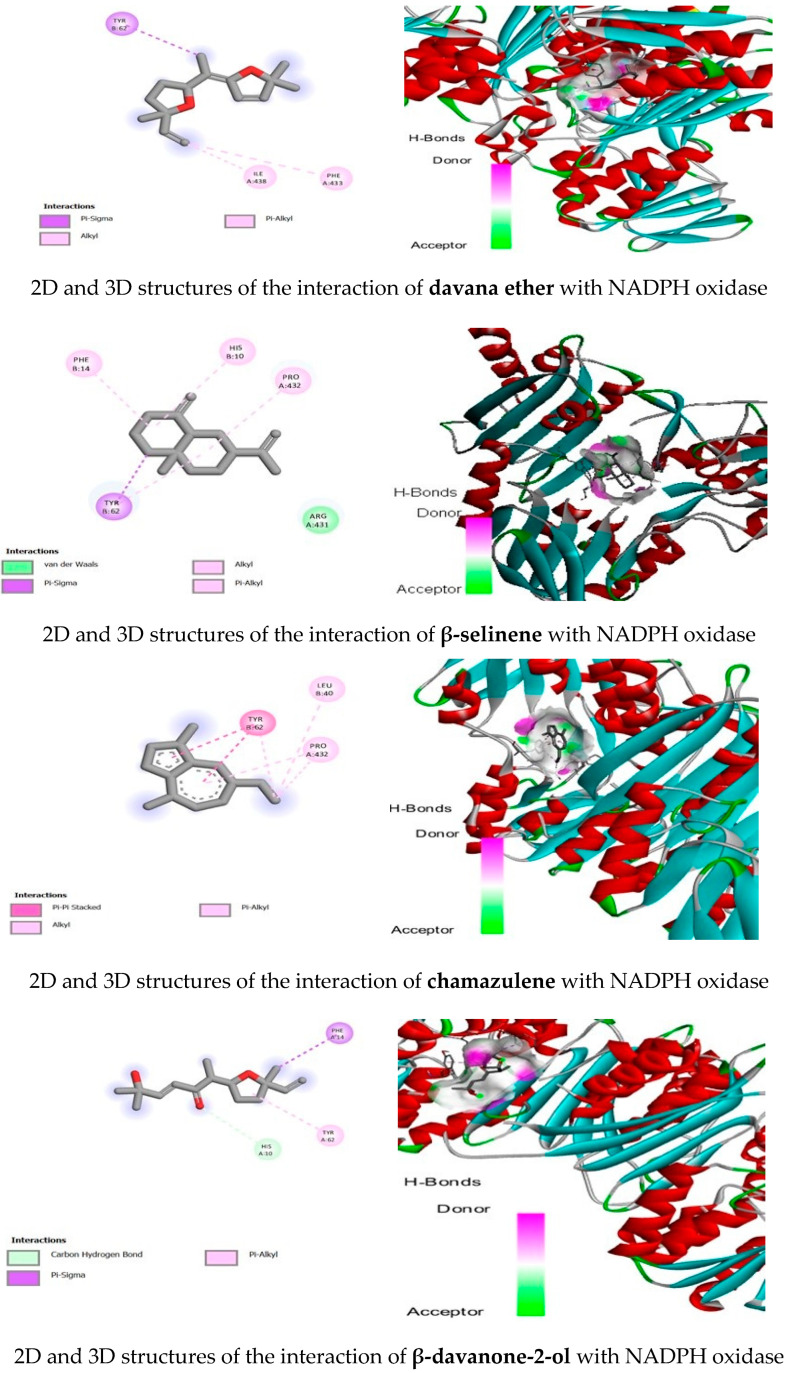

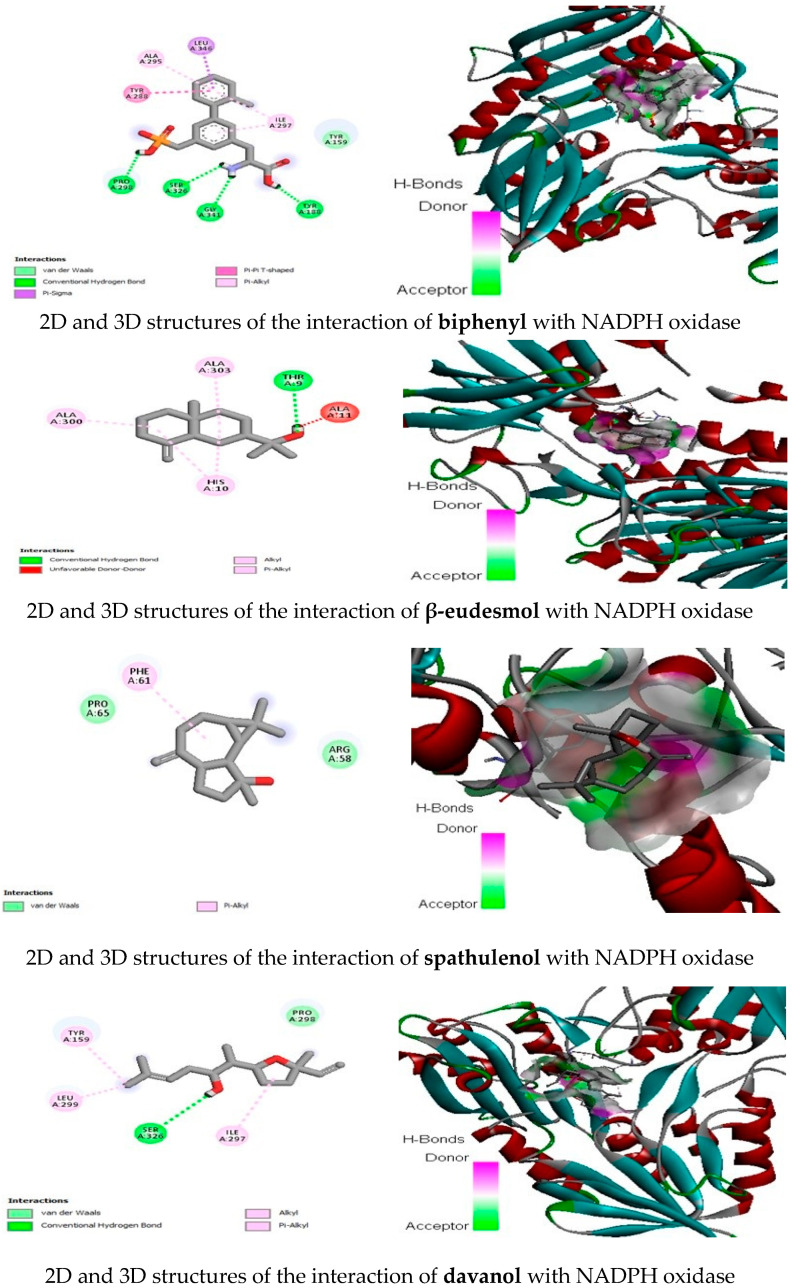
2D and 3D structures of the best docked compounds with the NADPH oxidase enzyme (with binding scores greater than −7.0).

To evaluate the potential contribution of *A. absinthium* EO to antioxidant activity, all compounds isolated by HD from fresh plant material and volatiles released from dried plant material sampled by HS-SPME, identified through GC-MS analysis, were docked against the NADPH oxidase enzyme alongside ascorbic acid (used as a standard). This enzyme has been linked to various diseases, including neurodegenerative disorders and cancer. Therefore, we selected it for current in silico molecular docking study [21]. Hydrophobic interactions, such as pi-alkyl and alkyl interactions, in addition to hydrogen bonds, play a crucial role in protein–ligand interactions and help improve stability. In summary, the compounds camphene, limonene, cis-linalool oxide (f), terpinolene, carvone, bornyl acetate, myrtanol acetate, ethyl hydrocinnamate, ethyl and methyl cinnamates, biphenyl, β-caryophyllene, β-farnesene, β-selinene, davana ether, (E)-nerolidol, cis-davanone, spathulenol, β-eudesmol, davanol, β-davanone-2-ol, and chamazulene may serve as potential inhibitors of the important enzyme NADPH oxidase, potentially contributing to the antioxidant properties of *A. absinthium*. These phytocompounds demonstrated superior binding interactions compared to the standard potent antioxidant compound derived from natural sources (ascorbic acid). Table 4 shows the predicted docking score results for the protein and ligand binding, while the two- and three-dimensional structures of compounds with binding scores greater than −7.0, along with the standard compound (ascorbic acid), are illustrated in Figure 1.

### 2.4. BOILED-Egg Model (In Vitro Pharmacokinetics Study)

**Figure 2:**
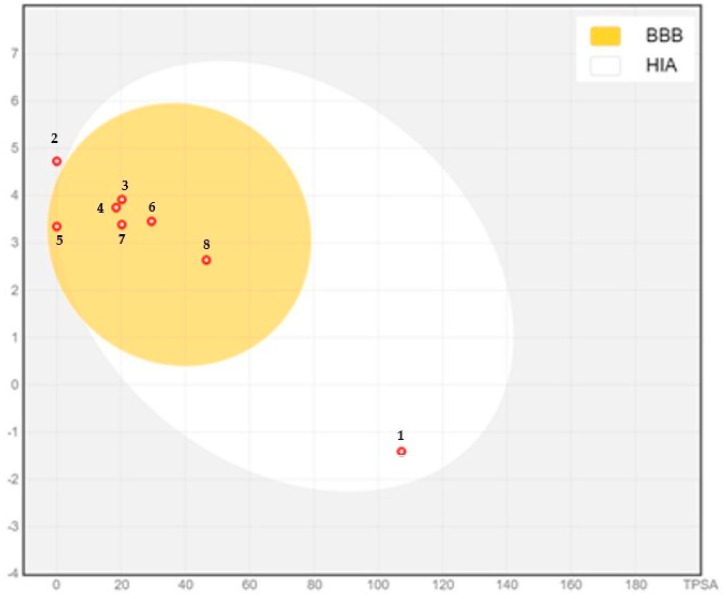
BOILED-egg plot of selected compounds with the best binding affinity: ascorbic acid (**1**), biphenyl (**3**), β-eudesmol (**4**), β-selinene (**2**), β-davanone-2-ol (**5**), spathulenol (**6**), davanol (**7**), and davana ether (**8**). BBB: blood–brain barrier; HIA: human gastrointestinal absorption.

Compounds having the best binding affinity, which were ascorbic acid, biphenyl, β-eudesmol, β-selinene, β-davanone-2-ol, spathulenol, davanol, and davana ether, were selected for the BOILED-Egg model. Ascorbic acid and β-selinene showed absorption from the gastrointestinal route, whereas all other compounds showed absorption from the blood–brain barrier (Figure 2).

## 3. Discussion

Essential oils are secondary products with scientifically proven biological properties used for centuries in treating many chronic diseases. Therefore, it is essential to study them in depth to understand their active compounds and physical and chemical properties, as well as their extraction methods. Research on *A. absinthium* EO has highlighted its complex composition and multiple biological activities, including antioxidant, antimicrobial, anti-inflammatory, antidepressant, antiulcer, and cytotoxic effects [17,19]. Key compounds such as thujone, myrcene, camphor, and linalool contribute significantly to these properties. Thujone is particularly effective against antibiotic-resistant pathogens, while camphor and myrcene aid in reducing inflammation and pain [19]. Additionally, camphor and linalool have shown anticancer potential by inducing apoptosis in liver and breast cancer cells. The EO’s antioxidant capacity, enhanced by phenolic compounds and flavonoids, also helps protect cells from oxidative stress, lowering the risk of chronic diseases. Together, these bioactive constituents make *A. absinthium* EO a potent and versatile therapeutic agent [17,18,19].

Solid-phase microextraction (SPME) has emerged as an effective sample preparation method, enabling the efficient isolation and concentration of analytes from complex matrices. One of the most common SPME approaches involves extracting directly from the headspace (HS) in equilibrium with the sample. HS-SPME offers superior enrichment, simplicity, versatility, and is often reusable, making it a solvent-free and environmentally friendly approach [12,13]. In this study, the essential oil was isolated from Saudi *A. absinthium* by the HD of fresh plant material and the volatiles released from dried plant material sampled by HS-SPME, followed by GC-MS analysis.

The results showed that HS-SPME identified a greater number of constituents belonging to various chemical classes (46 compounds) compared to HD (25 compounds), indicating that HS-SPME is more precise in preparing volatile samples from dried plant material with a higher concentration of compounds belonging to various chemical classes. All the identified compounds are described in Table 1. Cis-davanone was the most prevalent compound in HD EO, accounting for 60.2%, but only 18.0% in the HS-SPME-prepared EO. Similar high concentrations of cis-davanone have been reported in other Artemisia species, such as *A. chamaemelifolia* (57.3%) and *A. pallens* (53.0%) [23]. Camphor made up 26.4% of the HS-SPME-prepared EO and 6.3% of the HD-extracted EO. On the other hand, chamazulene, a bicyclic sesquiterpene derivative responsible for the dark blue color of the EO, was found in a higher concentration in HD essential oil (10.8%) while only showing 2.8% in the HS-SPME-prepared EO.

In the HD EO, the identified constituents were mainly categorized as the oxygenated sesquiterpene chemical class (64.3%), cis-davanone representing 60.2% of the total oil content, followed by chamazulene (10.8%). Meanwhile, oxygenated monoterpenes, represented by camphor and terpinen-4-ol, made up smaller portions (13.8%). In the HS-SPME-prepared EO, oxygenated monoterpenes constituted 42.0% of the total oil content, with camphor contributing 26.4%, while oxygenated sesquiterpenes and monoterpene hydrocarbons comprised 23.9% and 16.0%, respectively, exemplified by cis-davanone (18.0%) and limonene (3.3%).

Essential oils from *A. absinthium* located in Tunisia, Algeria, China, and the southern region of Saudi Arabia have been reported to have a dark blue color, which is attributed to the presence of chamazulene (around 8% or more). In contrast, essential oil from Lithuanian *A. absinthium* displays a dark brown hue due to compounds like trans-sabinyl acetate, β-pinene, trans-thujone, and myrcene, with chamazulene constituting only 1.5%. Similarly, EO from Tajikistan is yellow, with chamazulene being a minor component [18,24]. A previous study conducted by Mohammed H. (2022) on *A. absinthium* EO extracted via HD from Saudi species growing in the central region showed its major constituents to be cis-davanone (52.51%) and chamazulene (3.38%) [18]. In this study, the dark blue color of the Saudi *A. absinthium* collected from mountains in the southwestern region was due to the higher chamazulene content in the HD EO (about 10.8%). This variation in chamazulene concentration appears to explain color differences and may influence the biological activities of the essential oil [18]. The current study supports the existence of chemotype variations among *A. absinthium* from different regions worldwide and within the same country, with cis-davanone and chamazulene being the main constituents of EO in the Saudi species.

The importance of *A. absinthium* in this study lies in its demonstrated free radical scavenging potency and enzyme inhibition capabilities, confirming its role as a potent antioxidant and enzyme inhibitor remedy derived from a natural source.

The antioxidant properties of *A. absinthium* essential oils have been documented many times in the literature, though the methods used in previous studies differ from those applied in the current study, making direct comparisons difficult. The results in Table 2 show the strong antioxidant potential of the essential oil of *A. absinthium* obtained from southwestern Saudi Arabia and suggest that local environmental conditions play a major role in influencing the chemical composition and quality of the EO as well its biological potency.

The plant’s ability to neutralize free radicals, as shown through DPPH, ABTS, and FRAP assays, supports its potential use in oxidative-stress-related diseases. The total antioxidant capacity measured (821.8 mg ascorbic acid Eq/g EO) highlights the EO’s significant capacity to mitigate oxidative stress, which is associated with aging, cancer, and neurodegenerative disorders. This positions *A. absinthium* as a natural source of antioxidants that could be incorporated into therapeutic products targeting free-radical-induced damage.

The high antioxidant activity observed in this study may be due to the significant presence of cis-davanones, which accounted for 60.2% of the HD EO content [18]. In previous reports, plants rich in cis-davanones have been noted for their ability to scavenge free radicals [18,23,24]. Additionally, the higher levels of oxygenated monoterpenes and sesquiterpenes, such as camphor and cis-davanone, likely contribute to the enhanced antioxidant activity recognized in this study [18]. Chamazulene has demonstrated its effectiveness in many studies as an antioxidant [17,18]. In this study, this compound constituted about 10.8%, which is sufficient to show its free radical scavenging activity. Its effect may result from the presence of other compounds like cis-davanone and camphore in the essential oil that have a similar effect (i.e., synergistic effect), which enhances the efficacy of *A. absinthium* EO [17,18,19].

Antibiotics have significant effects against *Helicobacter pylori*, although 15.0% of patients do not respond to treatment. Natural extracts and their secondary metabolites like essential oils are effective alternatives to urease inhibition as a treatment for *H. pylori* infection [25]. This study showed that EO displays a significant inhibition of the urease enzyme (86.7%) compared to thiourea (91.7%). The GC-MS analysis indicated the presence of compounds belonging to different chemical classes that work synergistically, which is responsible for the effect of urease inhibition.

Additionally, the α-amylase enzyme causes the breakdown of glycogen and starch. Its inhibition could be effective in the treatment of obesity and diabetes. Due to polysaccharides, terpenoids, and glycosides, many plants have an α-amylase inhibitory potential [26]. This current work revealed that EO showed a significant inhibition of the α-amylase enzyme (81.8%), which is due to the presence of compounds, as assessed by the GC-MS profile, that work synergistically to achieve this interesting enzyme inhibition effect.

The essential oil demonstrated a substantial inhibition of the α-amylase and urease enzymes linked to diabetes/obesity and gastrointestinal ulcers, respectively. These findings suggest that the *A. absinthium* EO could play a role in managing diabetes by reducing postprandial glucose levels through α-amylase inhibition, while its urease inhibition points to potential antiulcer properties, particularly against *H. pylori*.

In vitro pharmacokinetics (PK) and pharmacodynamics (PD) studies are crucial for understanding the absorption, metabolism, efficacy, and toxicity of natural products and their metabolites. They help to optimize the bioavailability and therapeutic index and guide structural modifications while reducing the reliance on animal testing. These models provide early insights into drug interactions, toxicity, and mechanisms of action for effective drug development. To address these concerns, the current study performed in vitro experiments to evaluate the pharmacodynamics and pharmacokinetics properties of *A. absinthium* EO, utilizing in silico molecular docking and the BOILED-Egg model for the pharmacodynamics and pharmacokinetics assessments, respectively.

It is worth noting that there has been a growing trend in the use of computational techniques, such as molecular docking, to evaluate the interactions between natural products, their metabolites, and their biological targets. In this study, all the phytocompounds identified through GC-MS were tentatively analyzed using docking methods. Reactive oxygen species (ROS) are generated in the mitochondria through oxidative phosphorylation and NADPH oxidase, which also promotes abnormal cell proliferation and intracellular signaling processes. This phenomenon has been observed in various contemporary diseases. Consequently, inhibiting NADPH oxidase can demonstrate antioxidant and reactive oxygen scavenging capabilities [27]. To estimate the potential of *A. absinthium* EO in inhibiting NADPH oxidase enzymes and to correlate this with antioxidant activity, all compounds obtained from the essential oil isolated by the HD of fresh plant material and the volatiles released from dried plant material sampled by HS-SPME and analyzed by GC-MS, along with ascorbic acid as a reference standard, were subjected to docking against the NADPH oxidase enzyme.

The molecular docking findings showed that several essential oil active agents demonstrate stronger binding affinities to NADPH oxidase than the standard reference ascorbic acid (−6.1 kcal/mol). Notably, ligands such as davana ether (−7.9), chamazulene (−7.6), β-davanone-2-ol (−7.5), biphenyl (−7.4), β-selinene (−7.6), spathulenol (−7.3), β-eudesmol (−7.3), and davanol (−7.1) showed superior binding energies, indicating their potential as more potent inhibitors of NADPH oxidase. Hydrophobic interactions, including pi-alkyl and alkyl interactions, as well as hydrogen bonds, are essential for stabilizing protein–ligand interactions. Compounds such as biphenyl, β-caryophyllene, β-farnesene, β-selinene, davana ether, (E)-nerolidol, cis-davanone, spathulenol, β-eudesmol, davanol, β-davanone-2-ol, and chamazulene may act as potential inhibitors of the crucial oxidative enzyme NADPH and may contribute individually or synergistically to the antioxidant properties of *A. absinthium* EO.

We would like to highlight here that biphenyl is not traditionally classified as a typical phytocompound associated with *A. absinthium* EO profiles. Its detection in the oil sample prepared by HS-SPME but not in the essential oil extracted by HD suggests that it may be a byproduct of specific volatile-release mechanisms unique to the HS-SPME sampling method rather than being a primary constituent of the plant itself. This aligns with the findings where HS-SPME captures a broader spectrum of volatile compounds, including trace compounds or environmental contaminants that do not typically persist in distilled essential oils. However, biphenyl has been found to exhibit antioxidant properties in some contexts, though it is generally less potent compared to established natural antioxidants like flavonoids or phenolic compounds. Studies have shown that biphenyl and some of its derivatives can act as free radical scavengers or inhibitors of lipid peroxidation. However, its antioxidant potential may vary based on structural modifications and the specific environmental conditions under which it is studied [28].

In the context of *A. absinthium*, biphenyl might contribute minor antioxidant effects, but it would likely not play a central role compared to the more abundant compounds like davana ether, β-davanone-2-ol, and chamazulene, which are better known for their antioxidant efficacy.

These results suggest that the EO compounds may offer enhanced antioxidant and enzyme inhibitory effects, particularly in comparison to other natural and synthetic inhibitors. Given the enzyme’s role in oxidative-stress-related diseases like diabetes, these findings highlight the therapeutic promise of EO, warranting further in vivo studies to explore its potential for clinical applications like the streptozotocin (STZ)-induced diabetic rat model, which is a popular animal model for antidiabetic assays.

Furthermore, this study showed the components of *A. absinthium* EO exhibited diverse pharmacokinetics profiles as predicted by the BOILED-Egg model (Figure 2). Compound 1 (ascorbic acid, standard antioxidant natural-derived source) was predicted to be passively absorbed but unable to cross the blood–brain barrier (in the white) and PGP– (red dot), while compound 2 (β-selinene) was predicted to be neither absorbed via the GIT membrane nor brain-penetrant (outside the egg). Compounds 3 (biphenyl), 4 (β-eudesmol), 5 (β-dvanone-2-ol), 6 (spathulenol), and 7 (davanol) were predicted to penetrate the brain (in the yolk) and be actively effluxed (blue dot). Compound 8 (davana ether) demonstrated a strong ability to cross the blood–brain barrier (in the yolk) and was also subject to active efflux (blue dot).

In summary, this project highlighted the biological importance of *A. absinthium* EO as a valuable source of bioactive compounds. These compounds not only exhibit strong free radical scavenging potential but also offer significant enzyme inhibition effects, making this plant an exciting candidate for further pharmaceutical and therapeutic development to counter various degenerative diseases.

## 4. Materials and Methods

### 4.1. Plant Material

A total of 600 g of fresh aerial parts of *A. absinthium* were harvested at the flowering stage from the Al-Abadel Mountains located in the Jizan Province (southwestern region), Saudi Arabia (17° 0.110213 N, 43° 0.1559421 E), in September 2023. Then, about 10 gm was dried under normal air conditions, ground, and kept for essential oil sampling via the headspace solid-phase microextraction (HS-SPME) technique, while the remaining fresh plant material (around half a kg) was used for EO isolation via the HD method. The plant specimen was identified by Dr. Rajakrishnan Rajagopal, a plant taxonomist and botanist at the Science College Herbarium, King Saud University, in Riyadh, Saudi Arabia. It is archived in the herbarium department under voucher number KSU-5640.

### 4.2. Essential Oil Preparation from Dried Plant Material Using HS-SPME and GC-MS Analysis

The essential oil was sampled from the dried powder of the selected plant aerial parts using HS-SPME. The GC-MS and secondary metabolite identification were conducted as outlined in the methods described by Aati H et al. in the literature [29].

The powder (100 mg) was heated at 80 °C for 1 h in a headspace vial, with an SPME fiber (PDMS/DVB/Carboxen) (Merck, Product No. 57348-U, Darmstadt, Germany) collecting the volatiles at room temperature. The fiber was then desorbed at 250 °C in a GC injector for 1 min. GC-MS analysis was performed using an SE-52 capillary column (25 m in length, 0.5 mm internal diameter, 0.25 µm film thickness) (Macherey-Nagel™ 723312.25, Thermo Fisher Scientific, Monza, Italy) and a Thermo Fisher ISQ mass spectrometer in the electron ionization mode (70 eV), scanning a range of 40–500 amu, (ISQ™ EM, Thermo Fisher Scientific, Italy). Components were identified using the NIST Library (20, Software Version 2.4) spectral database and quantified based on the GC-MS peak areas.

### 4.3. Essential Oil Extraction by HD and GC-MS Analysis

Around half a kilogram of freshly sliced aerial parts of *A. absinthium* underwent hydrodistillation for 8 h using a Clevenger apparatus, following the standard procedure outlined in the European Pharmacopoeia (2005). The extracted EO was dissolved in petroleum ether and filtered through anhydrous sodium sulfate, yielding a deep blue EO with a 0.82% (*v*/*w*) yield. The EO was then stored in amber glass under nitrogen at 4 °C in the dark, pending the GC-MS analysis and antioxidant biological tests. An Agilent 7890B gas chromatograph, USA, equipped with an inert mass selective detector (5977B) and an SE-52 capillary column (25 m in length, 0.5 mm internal diameter, 0.25 µm film thickness) (Macherey-Nagel™ 723312.25, Thermo Fisher Scientific, Italy), was used for the analysis. A 2 µL sample was injected in the splitless mode, with the injector temperature set at 250 °C and the interface at 280 °C. The oven temperature program began at 100 °C for 0.5 min, then increased to 340 °C at a rate of 20 °C/min and was held for 1 min. Helium served as the carrier gas, with electron impact ionization at −70 eV in the full-scan mode. The total run time was 30 min, and compound identification was performed using the NIST Library 20.0 [30].

### 4.4. Biological Activities

#### 4.4.1. Antioxidant Activity

The antioxidant potential of EO was assessed using a total antioxidant assay, scavenging assays (ABTS and DPPH), and a reducing power assay (FRAP), as well as nitrite oxide scavenging methods.

- Total antioxidant activity assay (phosphomolybdenum method)

A previously established approach was modified to assess the total antioxidant activity [30]. In a 2 mL Eppendorf tube, 1.8 mL of reagent [0.6 M H_2_SO_4_, 4 mM (NH_4_)6MO_7_O_24_, 28 mM Na_2_PO_4_] and 200 µL of the EO solution were added. The tube was capped and left to incubate for around 90 min at 95 °C. At λ 695 nm, the absorbance was measured. Aliquots of ascorbic acid ranging from 35, 65, 125, 250, 500, 750, to 1000 µg·mL^−1^ in 5% DMSO were prepared for the calibration curve, and the total antioxidant activity of the EO was expressed as mg ascorbic acid equivalent per gram of essential oil (mg ascorbic acid Eq·gm^−1^ EO).

- Scavenging assay

The scavenging assay of EO was estimated by the ABTS assay (expressed in mg. ascorbic acid equivalent per gram of essential oil) and DPPH assay (expressed in mg. ascorbic acid equivalent per gram of essential oil). The methods for DPPH [31] and ABTS [32] were taken from the literature with slight modifications as described.

a. 2,2-Diphenyl-1-picrylhydrazyl (DPPH) assay

A total of 100 µL DPPH solution (0.03 mM in methanol) was taken in a microtiter plate; then, 100 µL of the EO solution was added. The mixture was incubated at an ambient temperature for half an hour in the dark. At λ 517 nm, an absorbance was recorded. The calibration curve of ascorbic acid was drawn at 70, 100, 150, 200, 250, 300, and 400 µg·mL^−1^ in methanol. The antioxidant potential was expressed as mg ascorbic acid equivalent per gram of essential oil (mg ascorbic acid Eq·gm^−1^ EO).

b. 2,20 -azino-bis (3-ethylbenzothiazoline-6-sulfonic acid (ABTS^+^) assay

A total of 1 mL of EO (1 mg.mL^−1^) was taken in a 20 mL vial which contained 2 mL of a mixture of the same volume of 2.45 mM potassium persulfate and 2.5 mM ABTS; then, the vials were incubated in the dark for half an hour. At λ 734 nm, absorbance was measured. The calibration curve of ascorbic acid was drawn using an ascorbic acid solution in 5% DMSO 5–80 µg·mL^−1^. The results were expressed in mg equivalents of ascorbic acid per gram of essential oil.

- Reducing antioxidant power (ferric reducing antioxidant power (FRAP)) assay

The reducing capacities of EO were determined by FRAP. Ferric reducing potential was assessed using a previously described method [32] with slight modifications. A total of 3 mL of EO solution (1 mg.mL^−1^) was taken in a vial; then, a 2 mL reaction mixture [0.3 M acetate buffer (pH 3.6, 20 mM ferric chloride: 40 mM HCl (10:1:1): 10 mM 2,4,6-tris(2-pyridyl)-s-triazine] was added. This solution was then incubated at an ambient temperature for half an hour, and at λ 593 nm, absorbance was measured. The calibration curve of ascorbic acid in methanol was constructed at 5–50 µg·mL^−1^. The results were expressed as a mg equivalent ascorbic acid per gram of essential oil (mg ascorbic acid Eq·gm^−1^ EO).

- Nitrite oxide scavenging

The nitric oxide scavenging effect of EO was assessed using the method described by Yen et al. (2001). In test tubes, 4 mL of EO solution at varying concentrations was mixed with 1 mL of a 25 mM sodium nitroprusside solution, and the tubes were incubated at 37 °C for 2 h. After incubation, 0.5 mL of the solution was withdrawn and diluted with 0.3 mL of Griess reagent (1% sulfanilamide in 5% H_3_PO_4_ and 0.1% naphthylethylenediamine dihydrochloride). The absorbance of the resulting chromophore, formed during the diazotization of nitrite with sulfanilamide and subsequent coupling with naphthylethylenediamine dihydrochloride, was measured at 570 nm and compared to the absorbance of standard sodium nitrite solutions that underwent the same treatment as the Griess reagent [33].

#### 4.4.2. Enzyme Inhibition Activities

##### Urease Inhibition Assay

The urease enzyme inhibition assay was conducted using a modified method from Bashir et al. (2021) [34]. In a 96-well ELISA microplate, 10 µL of a 0.5 mg/mL EO solution, 10 µL of a 1 M phosphate buffer solution (pH 7), and 10 µL of a 0.025% urease enzyme solution were combined and incubated for 15 min at 37 °C. Following this, 30 µL of a 2.25% aqueous urea solution was added as the substrate, and the mixture was incubated for an additional 15 min at 37 °C. The absorbance was measured at λ 630 nm as a pre-read. Next, 50 µL of an alkaline reagent (0.1% NaOCl + 0.5% *w*/*v* NaOH) and 30 µL of a phenol reagent (1% phenol *w*/*v* + 0.005% sodium nitroprusside) were added to the reaction mixture. The mixture was then re-incubated for 90 min at 37 °C, and the absorbance was measured again at λ 630 nm for the post-read. Thiourea was used as the standard, while the reaction mixture without the sample served as the control. The percentage inhibition of the urease enzyme was calculated using the formula:

**Inhibition (%) of urease enzyme** = 100 − [(Absorbance of Post-Read − Absorbance of Pre-Read)/Absorbance of control] × 100

##### α-Amylase Inhibition Assay

The method, which was previously described by Anyanwu et al. (2019) [35], was used to determine the inhibitory action of α-amylase. A total of 20 µL of starch (concentration 1% *w*/*v*), 20 µL of 6 mM NaCl, and 20 µL of EO was taken in an ELIZA microtiter plate; then, 25 µL of 0.02 M sodium phosphate buffer was added to this mixture and incubated at 37 °C for 5 min. Then, 15 µL of amylase solution was added into each well and incubated at 37 °C for the next 10 min. After that, 20 µL of 0.1 M dinitrosalicylic acid was added to terminate the reaction. Then, 100 µL of iodine reagent was added. Acarbose was used as a standard. The change in color was observed, and at λ 620 nm, absorbance was measured. Based on the amount of starch present, the mixture’s color varies. Dark blue is the color if there is an active inhibitor present. Yellow will be the color if there is no inhibitor. The color will be brownish if the starch is only partially degraded, indicating that the inhibitor is only partially active. The percentage inhibitions of the test solution were measured using the formula given below:

**Inhibition (%) of α-amylase enzyme** = [(Absorbance of control − Absorbance of sample)/Absorbance of control] × 100

### 4.5. In Silico Molecular Docking Study (NADPH oxidase Enzyme Inhibition), (In Vitro Pharmacodynamics Study)

The protein molecule nicotinamide adenine dinucleotide phosphate (NADPH) oxidase was obtained in the PDB format from the Protein Data Bank, with the PDB ID 2cdu. The protein was prepared using the Discovery Studio 2021 Client, where all chains except the A chain were removed, as well as any water molecules and ligands present. Polar hydrogen atoms were then added, and the final structures were saved in the Protein Data Bank (PDB) format. Secondary metabolites and standard ascorbic acid were downloaded from the PubChem database in the SDF (structure-data file) format. The prepared proteins were uploaded to PyRx software (version 0.8), where the enzyme was converted to a macromolecule. Ligands were also imported into PyRx, processed with Open Babel, minimized, and converted to the PDBQT ligand format. To validate the docking process, the co-crystallized ligand (ascorbic acid) was superimposed with extracted ascorbic acid from the crystal structure and redocked into NADPH oxidase crystal structures. Docking was performed with the grid box with the following dimensions: x: 64.5915, y: 63.3020, z: 69.0533. Finally, the interactions were visualized using Discovery Studio [29].

### 4.6. BOILED-Egg Model (In Vitro Pharmacokinetics Study)

The *A. absinthium* EO phytoconstituent PK were screened by selecting a BOILED-Egg model to determine their activity within the human body and was performed with the online SwissADME. The BOILED-Egg model enabled the connection between predicting blood–brain barrier (BBB) penetration and gastrointestinal (GI) absorption via analyzing the relationship between the total polar surface area (TPSA) polarity and (WlogP) lipophilicity properties. The yellow area (yolk) represented a high chance of brain penetration, while the white area indicated a high likelihood of passive absorption in the gastrointestinal tract. Additionally, points were marked red if predicted to be a non-substrate of P-gp (PGP−) and assigned blue if the compound was predicted to be actively effluxed by P-gp (PGP+) [36].

## 5. Conclusions

The present study conducted a comprehensive chemical analysis and assessment of the biological potential of Saudi *A. absinthium* EO. Additionally, this work clarifies the importance of phytochemical studies that could be used as important chemotaxonomic tools. *A. absinthium* EO was rich in constituents, which worked synergistically to achieve antioxidant and α-amylase and urease inhibitory activities, suggesting its potential for commercialization and use as a foundational element in synthesizing novel compounds aimed at treating enzyme-related disorders like diabetes. Future studies aimed at isolating novel bioactive constituents and elucidating the structures of pure compounds from EO could facilitate the development of new drugs with antimicrobial, antiulcer, antioxidant, antidiabetic, and antiproliferative properties derived from natural remedies, potentially being used alone or in combination with available synthetic drugs to overcome drug side effects caused by high dose administration. Moreover, in vivo pharmacological and toxicological evaluations could be carried out in the future to explore the efficacy of *A. absinthium* EO in treating diabetes, inflammatory diseases, proliferative disorders, wound healing, and other contemporary diseases associated with oxidative stress.

## Figures and Tables

**Table 1:** The essential oil composition of *A. absinthium* (essential oil was isolated by HD using fresh plant material and volatiles released from dried plant material were sampled by HS-SPME ^a^); the major constituents are highlighted in bold text.

			HS-SPME	HD
#	Compound ^b^	RI ^c^	%Conc.	
1	α-Thujene	927	1.7	1.4
2	α-Pinene	933	2.3	1.2
3	Camphene	948	3.0	tr
4	Sabinene	975	0.6	0
5	β-Pinene	977	0.2	0
6	Myrcene	993	1.2	tr
7	α-Phellandrene	1006	0.3	0
8	α-Terpinene	1018	0.8	tr
9	*p*-Cymene	1026	0.4	0
10	Eucalyptol	1030	0.2	0
11	Limonene	1034	3.3	tr
12	cis-Arbusculone	1051	0.1	tr
13	Artemisia ketone	1056	0.1	tr
14	γ-Terpinene	1060	1.4	1.3
15	4-Thujanol	1073	2.3	tr
16	2-Nonanone	1085	0.9	0
17	Cis-linalool oxide (f)	1089	0.1	0
18	Terpinolene	1090	0.5	0
19	Linalool	1098	3.7	tr
20	trans-Sabinene hydrate	1103	0.3	0
21	**Camphor**	1154	**26.4**	6.3
22	Camphene hydrate	1165	0.1	0
23	Borneol	1175	0.4	0
24	Terpinen-4-ol	1180	0.7	5.5
25	Dill ether	1187	0.5	0
26	α-Terpineol	1190	0.2	0
27	Dihydrocarvone	1245	1.0	0
28	Carvone	1265	2.0	0
29	Bornyl acetate	1288	4.2	2.1
30	Myrtanol acetate	1295	tr	0.5
31	Ethyl hydrocinnamate	1351	1.2	0
32	(Z)-Ethyl cinnamate	1378	1.4	0.7
33	(E)-Methyl cinnamate	1383	0.4	0
34	Biphenyl	1394	0.6	0
35	(E)-β-Caryophyllene	1420	1.0	0.2
36	(E)-β-Farnesene	1459	0.2	0.5
37	(E)-Ethyl cinnamate	1469	5.4	3.7
38	β-Selinene	1508	0.7	0
39	Davana ether	1513	0.2	1.3
40	(E)-Nerolidol	1568	4.6	2.8
41	**cis-Davanone**	1570	**18.0**	**60.2**
42	Spathulenol	1584	0.2	tr
43	β-Eudesmol	1598	0.2	tr
44	Davanol	1620	0.2	0
45	β-Davanone-2-ol	1725	0.6	0
46	**Chamazulene**	1741	2.0	**10.8**
**Oxygenated monoterpenes**			**42.0**	**13.8**
**Hydrocarbon monoterpenes**			**16.0**	3.9
**Oxygenated sesquiterpenes**			**23.9**	**64.3**
**Hydrocarbon sesquiterpenes**			1.9	0.7
**Other oxygenated chemical classes**			**11.3**	**15.7**
**Other hydrocarbon chemical classes**			0.6	0
**Total%**			**95.6**	**98.5**

^a^ Average values represent the peak area as a percentage of the total peak area for three replicate samples. ^b^ Compounds are listed in the order they were eluted from the column. ^c^ RI refers to the retention index. tr: compounds isolated in a percentage less than 0.05.

**Table 2:** Antioxidant potential of *A. absinthium* essential oil.

Sample	Total Antioxidant Capacity (mg Ascorbic Acid Eq·gm^−1^ EO)	Scavenging Assays (mg Ascorbic Acid Eq·gm^−1^ EO)		Reducing Power Assay (mg Ascorbic Acid Eq·gm^−1^ EO)	NO Assay
		DPPH	ABTS	FRAP	
EO	821.8 ± 1.4	535.1 ± 2.1	736.3 ± 2.8	121.3 ± 1.6	90.4 ± 1.4

Each test was repeated three times, and the results are reported as the mean ± standard deviation.

**Table 3:** Enzyme inhibition (%) of *A. absinthium* essential oil.

Sample	Urease Enzyme (% Inhibition)	α-Amylase (% Inhibition)
EO	86.7	81.8
Standard ^a/b^	^a^ 91.7	^b^ 90.8

All results are expressed in percentage; a: thiourea, b: acarbose.

**Table 4:** Phytoconstituent docking with binding scores at the active site of the NADPH oxidase enzyme.

Sr No	Compound Name	Binding Affinity (kcal/mol)
1	α-Thujene	−6.1
2	α-Pinene	−6.0
3	Camphene	−6.2
4	Sabinene	−6.0
5	β-Pinene	−5.9
6	Myrcene	−5.8
7	α-Phellandrene	−6.1
8	α-Terpinene	−6.0
9	p-Cymene	−6.0
10	Eucalyptol	−5.9
11	Limonene	−6.2
12	cis-Arbusculone	−5.2
13	Artemisia ketone	−5.4
14	γ-Terpinene	−6.0
15	4-Thujanol	−6.1
16	2-Nonanone	−5.1
17	Cis-linalool oxide (f)	−6.4
18	Terpinolene	−6.5
19	Linalool	−5.8
20	trans-Sabinene hydrate	−6.1
21	Camphor	−5.8
22	Camphene hydrate	−5.5
23	Borneol	−5.5
24	Terpinen-4-ol	−5.9
25	Dill ether	−6.5
26	α-Terpineol	−6.0
27	Dihydrocarvone	−6.0
28	Carvone	−6.2
29	Bornyl acetate	−6.5
30	Myrtanol acetate	−6.6
31	Ethyl hydrocinnamate	−6.4
32	(Z)-Ethyl cinnamate	−6.1
33	(E)-Methyl cinnamate	−6.4
34	Biphenyl	**−7.4**
35	(E)-β-Caryophyllene	−6.9
36	(E)-β-Farnesene	−6.8
37	(E)-Ethyl cinnamate	−6.6
38	β-Selinene	**−7.6**
39	Davana ether	**−7.9**
40	(E)-Nerolidol	−6.9
41	cis-Davanone	−6.9
42	Spathulenol	**−7.3**
43	β-Eudesmol	**−7.3**
44	Davanol	**−7.1**
45	β-Davanone-2-ol	**−7.5**
46	Chamazulene	**−7.6**
47	**Ascorbic Acid (Standard)**	**−6.1**

## Data Availability

Data are contained within the article or the Supplementary Materials.

## Supplementary Materials

The following supporting information can be downloaded at: https://www.mdpi.com/article/10.3390/ph17111460/s1, Figure S1. The GC-MS chromatogram of *A. absinthium* L. essential oil prepared by the HS-SPME technique revealed its composition, with the primary components identified at retention times of 25.8 and 45.1 min, corresponding to camphor and cis-davanone, respectively; Figure S2. The GC-MS chromatogram of *A. absinthium* L. essential oil isolated by the HD technique revealed its composition, with the primary components identified at retention times of 45.1 and 49.9 min, corresponding to cis-davanone and chamazulene, respectively.
