# Supplementary material for: Phytochemical Characterization Utilizing HS-SPME/GC-MS: Exploration of the Antioxidant and Enzyme Inhibition Properties of Essential Oil from Saudi Artemisia absinthium L
Source: Pharmaceuticals (Basel). 2024 Oct 31;17(11):1460. doi: 10.3390/ph17111460 (PMC11597886; doi:10.3390/ph17111460)
Supplement: Supplementary file 1 [file pharmaceuticals-17-01460-s001.zip › pharmaceuticals-3260242-supplementary.pdf]

Supplementary Materials

Phytochemical Characterization Utilizing HS-SPME/GC-MS: Exploration of the Antioxidant and Enzyme Inhibition Properties of Essential Oil from Saudi *Artemisia absinthium* L.

Hanan Y. Aati [1,*], Hala A. Attia [2], Arwa S. Alanazi [3], Luluh K. AL tamran [3] and Juergen K. Wanner [4]

[1] Department of Pharmacognosy, College of Pharmacy, King Saud University, P.O. Box 2457, Riyadh 11451, Saudi Arabia; hati@ksu.edu.sa

[2] Department of Pharmacology and Toxicology, College of Pharmacy, King Saud University, P.O. Box 2457, Riyadh 11451, Saudi Arabia; hsalem@ksu.edu.sa

[3] College of Pharmacy, King Saud University, P.O. Box 2457, Riyadh 11451, Saudi Arabia; 441200341@student.ksu.edu.sa (A.A.); 441200557@student.ksu.edu.sa (L.A.t.)

[4] Kurt Kitzing Co., Hinterm Alten Schloss 21,D- 86757 Wallerstein, Germany; juergen.wanner@kurtkitzing.de

* Correspondence: hati@ksu.edu.sa

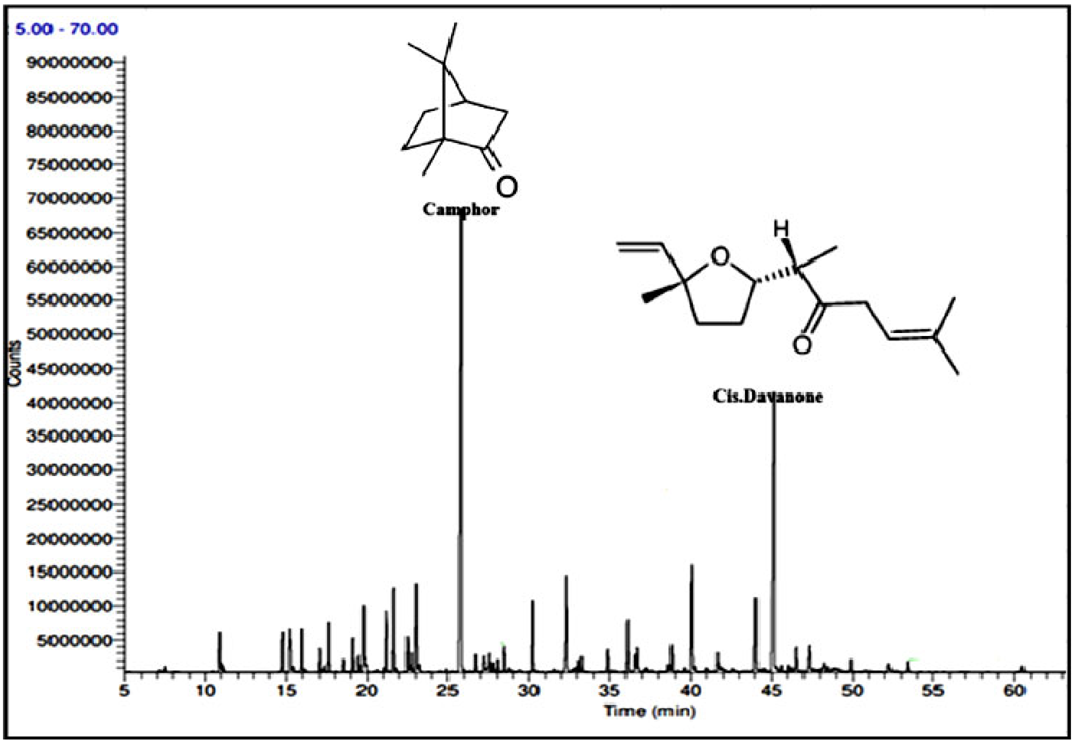

Figure S1. The GC-MS chromatogram of *A. absinthium* L. essential oil prepared by HS-SPME technique, revealed its composition with the primary components identified at retention times of 25.8 and 45.1 min, corresponding to camphor and cis-davanone, respectively.

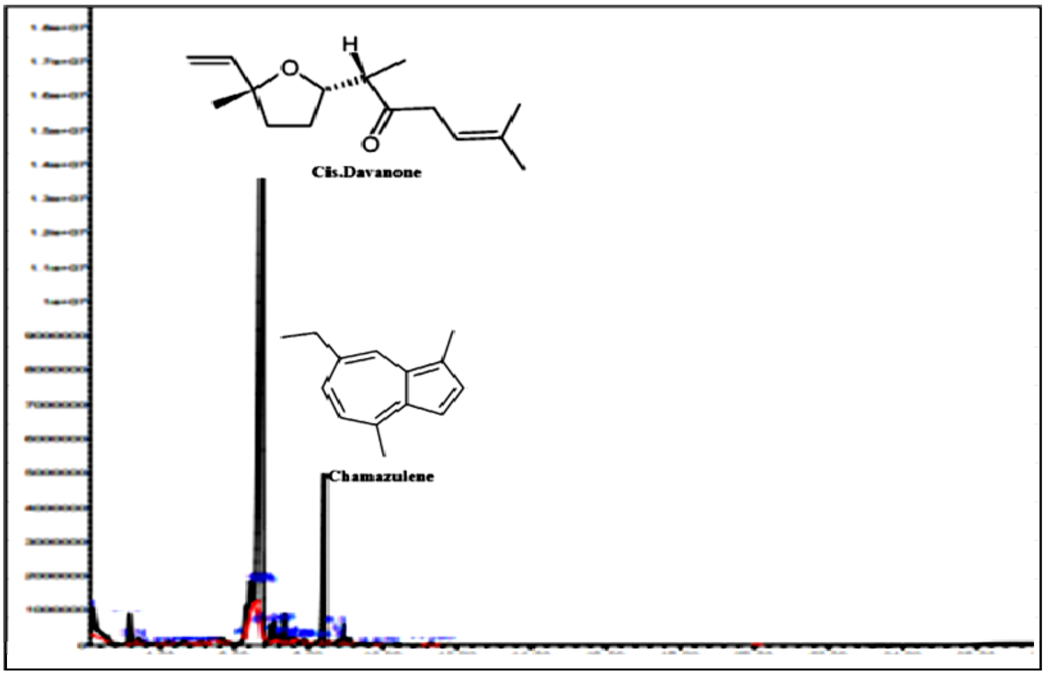

Figure S2. The GC-MS chromatogram of *A. absinthium* L. essential oil isolated by HD technique, revealed its composition with the primary components identified at retention times of 45.1 and 49.9 min, corresponding to cis-davanone and chamazulene, respectively.
